# Development of a new bicistronic retroviral vector with strong IRES activity

**DOI:** 10.1186/1472-6750-6-4

**Published:** 2006-01-12

**Authors:** Patrick Martin, Olivier Albagli, Marie Christine Poggi, Kim E Boulukos, Philippe Pognonec

**Affiliations:** 1 CNRS UMR6548, Parc Valrose, Université de Nice Sophia Antipolis, Nice, France; 2 INSERM U362, Institut Gustave Roussy, Villejuif, France

## Abstract

**Background:**

Internal Ribosome Entry Site (IRES)-based bicistronic vectors are important tools in today's cell biology. Among applications, the expression of two proteins under the control of a unique promoter permits the monitoring of expression of a protein whose biological function is being investigated through the observation of an easily detectable tracer, such as Green Fluorescent Protein (GFP). However, analysis of published results making use of bicistronic vectors indicates that the efficiency of the IRES-controlled expression can vary widely from one vector to another, despite their apparent identical IRES sequences. We investigated the molecular basis for these discrepancies.

**Results:**

We observed up to a 10 fold difference in IRES-controlled expression from distinct bicistronic expression vectors harboring the same apparent IRES sequences. We show that the insertion of a HindIII site, in place of the initiating AUG codon of the wild type EMCV IRES, is responsible for the dramatic loss of expression from the second cistron, whereas expression from the first cistron remains unaffected. Thus, while the replacement of the authentic viral initiating AUG by a HindIII site results in the theoretical usage of the initiation codon of the HindIII-subcloned cDNA, the subsequent drop of expression dramatically diminishes the interest of the bicistronic structure. Indeed, insertion of the HindIII site has such a negative effect on IRES function that detection of the IRES-controlled product can be difficult, and sometimes even below the levels of detection. It is striking to observe that this deleterious modification is widely found in available IRES-containing vectors, including commercial ones, despite early reports in the literature stating the importance of the integrity of the initiation codon for optimal IRES function.

**Conclusion:**

From these observations, we engineered a new vector family, pPRIG, which respects the EMCV IRES structure, and permits easy cloning, tagging, sequencing, and expression of any cDNA in the first cistron, while keeping a high level of expression from its IRES-dependent second cistron (here encoding eGFP).

## Background

Expressing two distinct coding sequences under the control of a unique promoter is of great interest to molecular and cellular biologists. Since both proteins are under the control of the same promoter, detection of the product encoded by the second cistron is the insurance that the first cistron is also being expressed. One can take advantage of bicistronic expression to select clones based on a resistance encoded by the second cistron [1], as well as sorting cells upon their GFP expression status. Polycistronic expression is also of potentially great interest when simultaneous expression of two different proteins is required for development of new gene therapy approaches [2]. Several commercially available bicistronic vectors are based on the Internal Ribosome Entry Site (IRES) from the Encephalomyocarditis virus (EMCV), in which the AUG has been modified to a HindIII site. This modification facilitates the cloning of the IRES-controlled cDNA, and insures that the initiation codon stems from the coding sequence inserted downstream of this cloning site. This avoids the N-terminal addition of amino acids derived from the junction of the IRES to the coding sequence of interest. However, previously published observations [3,4] demonstrated that this AUG is actually the most efficiently recognized by the translation machinery in the EMCV IRES. We compared the expression of a full-length cDNA cloned either in the HindIII-modified EMCV IRES (the ATG initiation codon being that of the cDNA), or downstream of the original unmodified EMCV initiation codon (which is then used as the initiation codon). We observed that expression of the second cistron in the HindIII-modified vectors can be as much as 10 fold lower than the expression of the same cistron whose translation initiates at the original, non-modified EMCV initiation codon.

In view of this dramatic decrease of IRES activity, we developed a new and convenient set of eukaryotic bicistronic expression vectors that respects the original EMCV initiation environment and fulfills several important needs: ease of cloning, sequencing possibilities of the cloned inserts using universal primers, high level of plasmid DNA production, possibility of epitope tagging for immunological tracking of expressed inserts, possibility of production of retroviral particles for retroviral transduction, and easy monitoring of expression through the concomitant synthesis of a fluorescent protein (GFP).

## Results

### Selection of an optimal IRES sequence

We observed that the expression of coding sequences derived from the second cistron of bicistronic vectors built on the IRES derived from the EMCV genome varied widely depending upon the vectors used. Specifically, the intensity of fluorescence driven by the second cistron of the pAP2 vector [5] was consistently much weaker than the intensity of fluorescence driven by the second cistron of the pMigR vector [6], despite the fact that both utilize the EMCV-derived IRES. We sequenced the IRES from both vectors and observed that the ATG codon that had been previously shown to be used as the initiating codon in EMCV [3], has been modified to a HindIII site in the pAP2 vector, while remaining untouched in the pMigR vector (This AUG is at position 860 in the 7861 bp EMCV sequence deposited in Genbank under the accession X74312, see Table 1). In order to test the possibility that this change could be responsible for the low eGFP expression level seen in pAP2, we engineered pMigR-derived vectors identical in sequences, except for the EMCV IRES initiation codon flanking region: pMigR-ATG contains the wild type IRES sequence, while pMigR-Hd contains the HindIII modified EMCV IRES. We inserted the eGFP coding sequence with its own ATG downstream of these IRESes, so that the fluorescence directly reflects the IRES activity.

293T cells were transfected with the same amount of the different vector DNAs, and were subsequently subjected to FACS analysis. As indicated in Table 1, fluorescence intensity was reproducibly much weaker from the HindIII-modified IRES (pMigR-Hd) compared to the expression from its wild type counterpart (pMigR-ATG) or the original pMigR, despite that in both cases their transcription originated from the same LTR promoters. The HindIII-modification of the EMCV IRES Initiation codon, in agreement with early reports on its structure and function [3], is thus responsible for an over 10 fold decrease in expression (14 versus 234 peak values). A similar analysis, performed with the same vectors containing an additional cDNA cloned into the first cistron (pMigR-Hd-BAZF and pMigR-ATG-BAZF), gave very similar results (Table 1). This confirms that the difference in the efficiency of expression of the second cistron is solely the result of differences in the IRES AUG initiation codon flanking sequences. The proportion of GFP positive cells is comparable in all cases, indicating that the strong difference of peak values is not due to differences in transfection efficiency.

**Figure 1:**
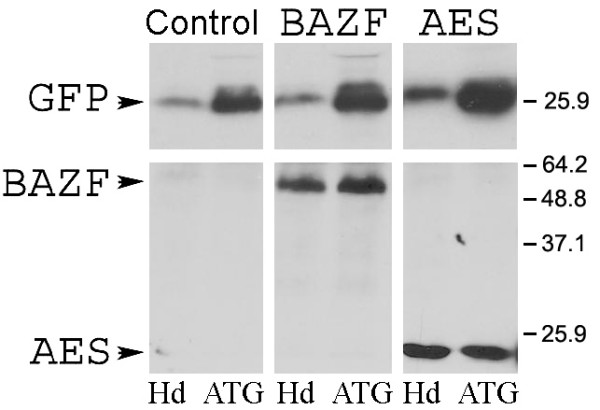
**Western blot analysis of HEK 293T cell lysates**. Cells were transfected with pMigR-Hd and pMigR-ATG (Control), or pMigR-Hd-BAZF and pMigR-ATG -BAZF (BAZF), or pMigR-Hd-AES and pMigR-ATG-AES (AES). Hd: pMigR-Hd based vectors, ATG: pMigR-ATG based vectors. GFP, BAZF and AES: translation products of the corresponding cDNAs. Molecular weight markers are indicated.

To rule out that the differences in fluorescence intensities would result from differences in promoter activities rather than from a decrease in the expression of the IRES-controlled fluorescent protein, we performed Western blot analyses with lysates prepared from pMigR-Hd-BAZF and pMigR-ATG-BAZF expressing cells, as well as from pMigR-Hd-AES and pMigR-ATG-AES expressing cells. BAZF and AES are two proteins of different molecular weights and structures, both tagged with the same HA epitope. Their expression thus reflects LTR promoter activities, while eGFP expression is a reflection of IRES activities. As can be seen in Fig 1, while the signals corresponding to the proteins under the direct control of the LTR promoter remain constant independent of the IRES present, the eGFP signal is strikingly decreased in the case of the HindIII-modified IRES.

Altogether, these experiments confirm that the integrity of the initiating AUG of the EMCV IRES is absolutely required for optimal activity, and that the modification brought by the creation of a HindIII site results in a severe loss of protein expression. It thus appears that when a high expression of the IRES-controlled cistron is needed, one should verify the integrity of the EMCV IRES present in the expression vector used, and shift to a vector containing the wild type form of the EMCV IRES.

### Engineering of a new and convenient retroviral IRES- containing vector, pPRIG

In order to palliate the problems stemming from the use of HindIII-modified IRES-containing expression vectors, we set up to develop a new vector that would permit i) easy directional cloning and sequencing of most inserts; ii) possible HA-tagging of the expressed proteins; iii) easy detection of the transduced cells through high expression of the IRES-controlled eGFPand iv) production of retroviral particles if needed. We called this new vector series pPRIG, for plasmid Polylinker Retroviral IRES GFP.

#### Bidirectional polylinker design

We designed a new polylinker which allows for the bidirectional cloning of a wide range of fragments. We used a strategy in which any site localized on one side of the polylinker has a matching counterpart on the other extremity. Altogether, our polylinker contains 20 unique restriction sites, including 15 sites allowing bidirectional cloning (Table 2). For example, an EcoRI/NotI DNA fragment can be inserted directly in the polylinker either by opening it with EcoRI and NotI, or in the reverse orientation by digesting the polylinker with NotI and MfeI, which is compatible with EcoRI. Our design permits the bidirectional cloning of up to 54 distinct site combinations, compared to, for example, the pBSK linker family which allows for only 7 such bidirectional clonings through the NotI/Bsp120I compatible sites. To our knowledge, our polylinker structure is the first one proposed with such a high number of possible bidirectional clonings.

#### N-terminal HA-tagging

**Figure 2:**
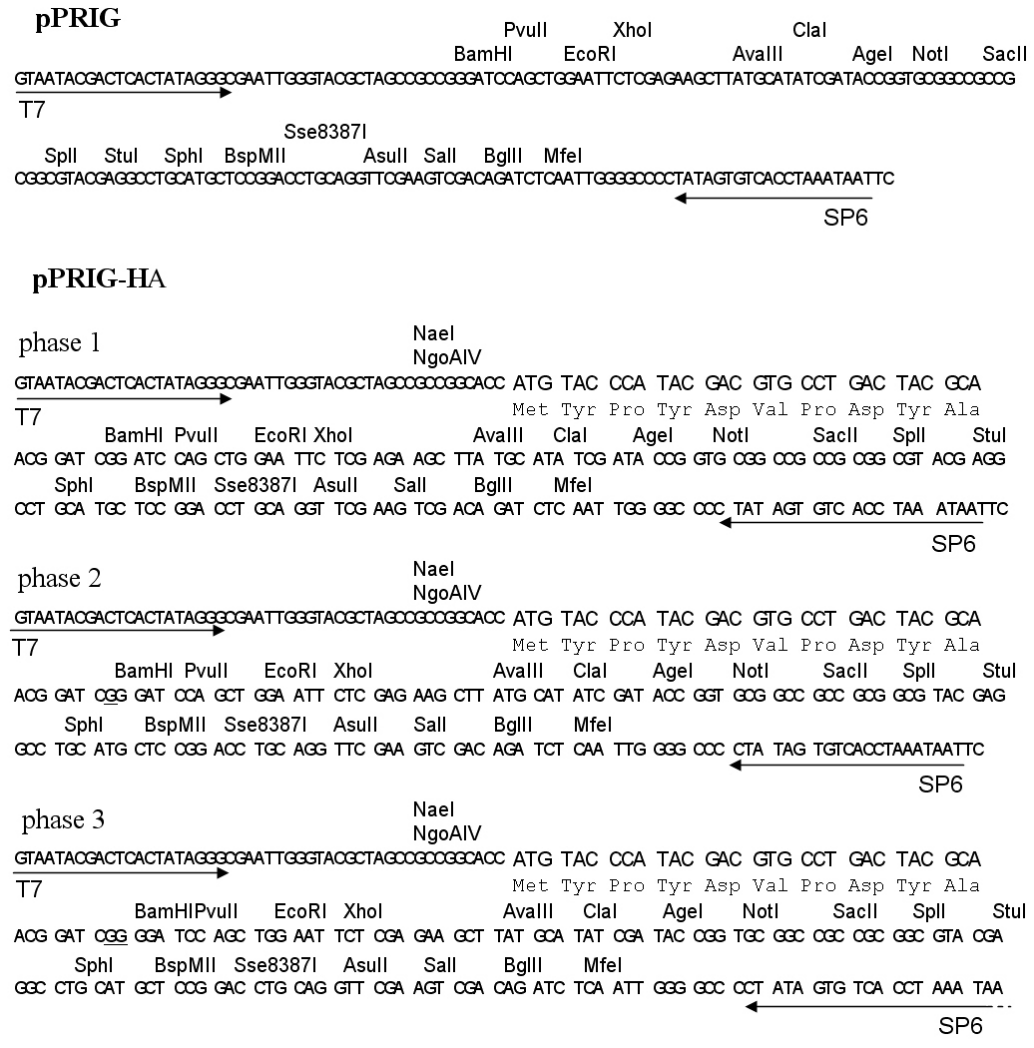
**Structure of the four pPRIG vector polylinkers**. pPRIG is the prototype polylinker, while the three pPRIG-HA vectors contain additional 1-nucleotide frame-shifted coding sequences for HA-tagging 5' of the polylinker sequence. Any insert can thus be cloned either in-frame downstream of this HA-tag, or directly into the pPRIG, without the HA-tag.

The pPRIG vector is designed to allow the fusion of an HA-tag in the N-terminal part of the protein of interest if needed. This way, the translation product of the cloned insert can be monitored through immunological approaches (immunolocalization, ELISA, Western blotting, immunoprecipitation...). To facilitate in phase-cloning, 3 versions have been developed (pPRIG-HA1, 2 and 3, see Fig. 2), each with a one nucleotide shift upstream of the stretch coding for the classical HA-tag. Thus, depending upon the reading frame of the insert of interest, either pPRIG-HA1, pPRIG-HA2 or pPRIG-HA3 can be used. Fig. 2 illustrates the 4 different polylinker structures available in the pPRIG vector family.

#### Direct sequencing with T7 and SP6primers

Flanking of the polylinker with T7 and SP6 universal primer sites makes sequencing of any insert straightforward. Our vectors are simultaneously cloning, expression and proviral vectors therefore no additional subcloning into other vectors is required. Sequencing of the insert of interest with T7 and SP6 primers allows for the verification of the integrity of the insert as well as the coherence of the coding frame following *in vitro *transcription/translation. Amplification of the plasmid is all that is needed to obtain material for functional assays in cells. The backbone of the vectors is derived from the pUC vector series, which insures a high DNA yield during plasmid amplification. We routinely obtained production of at least 5 mg/l of culture medium (LB-50 μg/ml Ampicillin) with all of our constructs, reaching up to 20 mg/l with some, as well as with the empty vectors. The production differences observed are likely to reflect the impact of the cloned inserts on plasmid amplification.

#### Retroviral backbone

Transfection efficiency remains one of the most severe limitations in the functional analysis of exogenously expressed cDNAs. This is why we chose to build the pPRIG vector on a retroviral backbone, which allows for the production of viral particles when needed, in order to obtain a high percentage of expressing cells. As a starting backbone for our pPRIG constructs, we used the pAP2 construct, which was kindly provided to us by Dr. J. Galipeau [5]. This construct contains a CMV promoter, which efficiently drives the expression of the bicistronic RNA following transfection in cells and which is replaced by an LTR when used in a retroviral context, due to the 3' LTR duplication upon reverse transcription.

**Figure 3:**
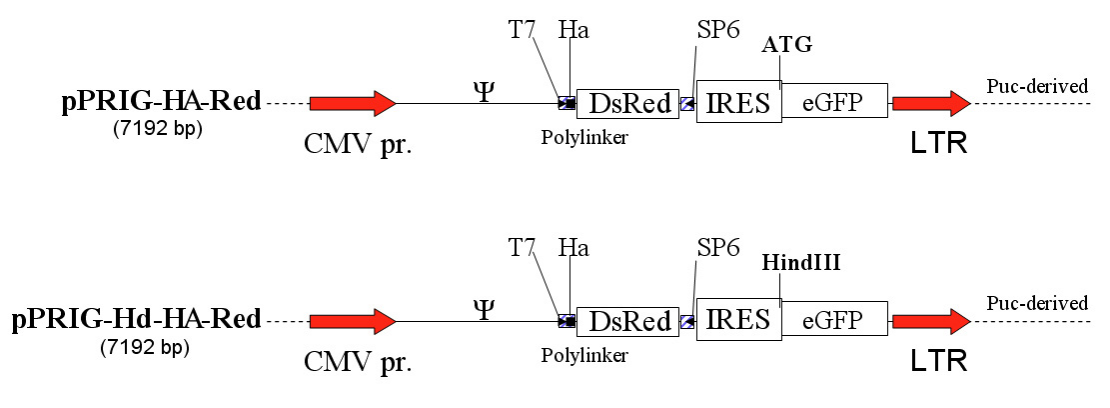
**Schematic representation of the pPRIG-HA-Red and pPRIG-Hd-HA-Red retroviral vectors**. Top drawing illustrates pPRIG-HA-Red, in which the coding sequence of DsRed has been cloned in the polylinker, while pPRIG-Hd-HA-Red is the same exact construct except that the IRES initiation codon is modified into a HindIII site (see Table 1). The psi letter (Ψ) is the MLV encapsidation sequence, the CMV promoter drives the expression of the bicistronic RNA, the hatched boxes flanking DsRed correspond to the polylinker, encompassing the T7 and SP6 sequences and the HA coding sequence, the IRES corresponds to the EMCV-derived IRES fragment, eGFP is the coding sequence for green fluorescent protein used to track the first cistron expression, and the LTR corresponds to the MLV long terminal repeat. The hatched line symbolizes Puc-derived plasmid backbone.

**Figure 4:**
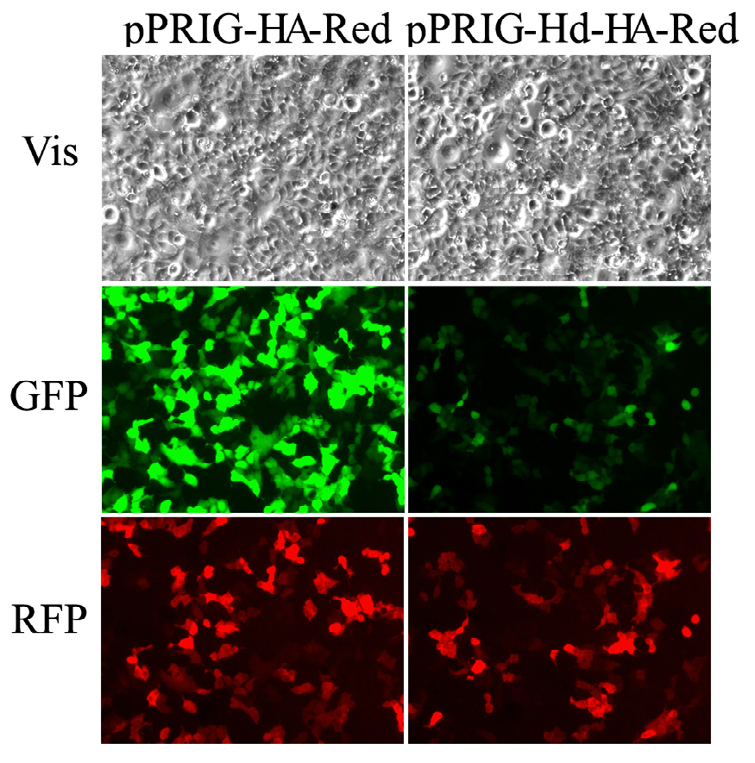
**Comparison of transient pPRIG-HA-Red and pPRIGw-HA-Red expressions**. HEK 293T cells were transfected with each vector, and the cells were photographed 48 h later. Vis: phase contrast image. Exposure time is the same for the two GFP images, and for the two RFP images. Fluorescence signals are thus a direct reflection of protein expression levels.

Since it is important to monitor the percentage of the population actually expressing the vector following transfection or transduction, we inserted the eGFP coding sequence downstream of the IRES. We then confirmed that the large difference in IRES dependent-eGFP expression detected by FACS and Western analyses using the pMigR constructs remains true with the pPRIG vector. To that end, we reintroduced a HindIII-modified IRES in the pPRIG vector, and cloned DsRed, which codes for a red fluorescent protein (RFP), under the direct control of the CMV promoter, creating the pPRIG-Hd-HA-Red. We also constructed the equivalent wt IRES-containing plasmid: pPRIG-HA-Red. The schematic representation of these vectors is depicted on Fig 3. The red and green fluorescences were followed under the microscope (Fig. 4). While the first cistron activity, reflected by the RFP signal, remains similar in both the wild type and the HindIII-modified IRES containing constructs, the eGFP signal was again strongly diminished when the IRES was modified by the insertion of the HindIII site. The relatively weak signal observed for DsRed is inherent to the weaker fluorescence of DsRed as compared to eGFP.

### Transduction assay

**Figure 5:**
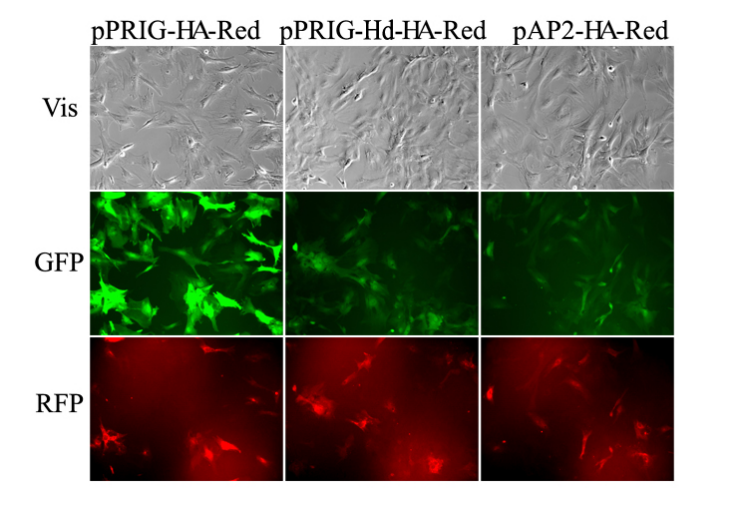
**Comparison of stable pPRIG-HA-Red, pPRIG-Hd-HA-Red and pAP2-HA-Red expressions**. HEK 293T cells were cotransfected with each vector and helper constructs. Viral supernatants were collected after 2 days and applied to primary rat embryo fibroblasts. The REF cells were photographed two weeks post-transduction. Vis: phase contrast image. Exposure time was the same for the three GFP images, as well as for the three RFP images. Fluorescence intensities are thus a direct reflection of protein expression levels.

**Figure 6:**
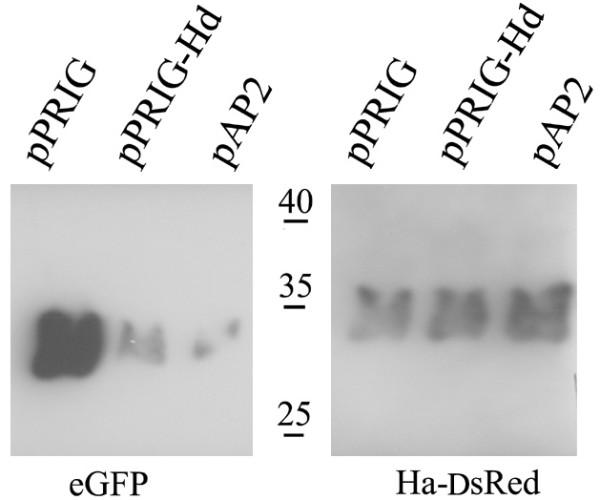
**Comparison of the pPRIG-HA-Red (pPRIG), pPRIG-Hd-HA-Red (pPRIG-Hd) and pAP2-HA-Red (pAP2) GFP and RFP protein expression by Western blot analysis**. Equivalent amounts of cell lysates containing the eGFP and HA-DsRed expressed from the indicated vectors were loaded in duplicate, ran on SDS-PAGE, and analyzed with an anti GFP serum (left panel) and an anti-HA serum (right panel).

We cotransfected 293T cells with pPRIG-HA-Red and proviral helper vectors to produce retroviral particles. The viral supernatant was then used to transduce exponentially growing primary rat fibroblasts (REF), and fluorescence was observed two weeks post transduction. A parallel experiment was performed with the pAP2-HA-Red, as well as with the pPRIG-Hd-HA-Red, and expression from each LTR (reflected by the HA-Red signals) was compared with the expression stemming from the different IRESes (reflected by the eGFP signals). First, we compared the viral titers obtained with the three constructs. To that end, serial dilutions of the supernatants were used to transduce REF. Monitoring of the fluorescence in transduced cells indicated a titer in the range of 1 × 10^6 ^per ml for all three vectors (data not shown), indicating that the polylinker and/or the modification of the IRES does not affect viral activity. Second, we observed that HA-Red expression was similar among the three vectors, indicating that the IRESes and/or polylinkers did not affect the expression driven by the LTR (Fig. 5). Third, and in good agreement with the results obtained in our transient HEK 293T transfections, IRES-dependent eGFP expression from the pPRIG vector was much more efficient than from pAP2 or pPRIG-Hd, as illustrated in Fig. 5. The differences in fluorescence were then monitored by Western analysis. Lysates from cells expressing these constructs were loaded in duplicate and run by SDS-PAGE, then subjected to either an anti-GFP antibody or an anti-HA antibody. Strikingly, we could barely detect the eGFP signal in the pRIG-Hd and pAP2 constructs, while the pPRIG displayed a strong signal (Fig. 6). In contrast, the HA-Red signal was comparable among the three vectors, indicating that the first cistron is similarly expressed in all three constructs, whereas expression of the second cistron is highly dependent upon the IRES integrity.

## Discussion

Extensive research over the last 15 years has led to significant advances in deciphering the molecular mechanisms involved in IRES function. All studies pointed to the involvement of secondary structures in the recognition of functional ribosome entry sites, as well as the importance of the distance between the polypyrimidine track and the actual initiating AUG. In the case of the Encephalomyocarditis virus-derived IRES, the second AUG located 22 nucleotides downstream of the UUUCC sequence, present in the polypyrimidine-rich track at the 3' of the IRES, has been identified as the authentic viral initiation codon [3,4,7]. Some reports indicated that insertion of a spacer up to 95 nucleotides between the 3' end of the IRES and the ORF of interest was not deleterious to the second cistron expression, provided that no secondary structure or out of frame initiation codon were present within that spacer [8]. Several constructs have been subsequently developed in which the original AUG (gat gat aat **ATG **gcc aca) was modified to a HindIII site (gat gat **AAGCTT **gcc aca), with the idea that cloning of a cDNA in this HindIII site would result in a further scanning of the ribosomes down to the AUG found in the cloned cDNA where initiation would occur. In this report we showed that despite its popularity among scientists wishing to use an IRES-containing expression vector, this modification present in many commercial vectors actually results in a dramatic decrease in the expression of the IRES-controlled coding sequence as compared to that observed with the wild type IRES. Earlier studies demonstrating that the EMCV IRES possesses a scanning independent fixed location AUG [3], together with the observations reported here, clearly support that the translational initiation is much more efficient from the natural (non mutated) IRES than from a HindIII mutated counterpart. This confirms that the IRES and the initiation codon are not completely independent modules that can be freely separated and remixed with heterologous sequences, as is often done. In our studies, we used the eGFP sequence as a reporter, whose initiator AUG is located 12 bases downstream of the engineered HindIII site (Table 1). Such a short distance is nevertheless sufficient to greatly affect the eGFP expression, despite the conservation of the optimal eGFP AUG context (acc AUG gug). Indeed, FACS analyses indicated a more than 10 fold difference in eGFP peak intensity. In addition, Western blot analysis of eGFP expression following viral transduction using HindIII-modified vectors displayed a weak eGFP signal, whereas wild type IRES resulted in a strong eGFP signal. As a control, both wild type and HindIII-modified constructs expressed HA-DsRed protein independent of the different IRESes, and comparable amounts of HA-DsRed were detected by Western blot analysis. In view of these results, we constructed a new vector family that allows for a wide range of applications, while keeping a strong IRES-dependent expression. This vector series has been called pPRIG, for plasmid Polylinker Retroviral IRESGFP. Due to a newly designed type of polylinker, directional cloning of a wide range of inserts is greatly facilitated, and HA-tagging of the insert of interest is possible if needed, depending upon which pPRIG vector is selected. Furthermore, the presence of T7 and SP6 primer sequences renders the sequencing of the cloned inserts straightforward, while allowing for the verification of insert expression via *in vitro *transcription/translation. Finally, our vectors are built on a retroviral backbone, allowing highly efficient cell transduction if needed. These vectors will be made freely available to the scientific community upon request.

## Conclusion

A survey of commonly used EMCV IRES-derived bicistronic vectors indicated that many of them, including several commercial ones, have their 11^th ^AUG modified to a HindIII site. In this work we confirm previous results demonstrating that the EMCV IRES predominant AUG (11^th^) cannot be modified without strongly affecting the overall efficiency of the IRES-dependent expression. Consequently, we show that all HindIII modified bicistronic vectors are very ineffective for their IRES-dependent expression. We developed a new family of expression vectors, called pPRIG, in which the structure of the EMCV IRES is left untouched. We verified that the IRES-dependent eGFP expression is much stronger than the HindIII modified IRES, and remains strong for weeks, even in a retroviral context. The pPRIG also contains a new type of polylinker that allows bidirectional cloning. In addition, this vector family permits easy HA-tagging, sequencing and in vitro expression of the insert, as well as the production of retroviral particles if needed.

## Methods

### Constructs

All constructs were made using classical molecular biology techniques, as described in [9]. A detailed construct strategy is available upon request, but briefly, the four pPRIG vectors were constructed as follows. The pPRIG-Hd-HA vectors were constructed from the pAP2-IRESeGFP, provided by Dr. J. Galipeau [5]. The EcoRI, NotI, PvuII, SalI, SphI, SseI restriction sites were removed from the vector in order to include them in a newly developed Multiple Cloning Site (MCS). A sequence encompassing a T7 bacterial promoter and an HA tag (followed by BamHI and EcoRI sites) was inserted between the end of delta-gag and the beginning of the IRES. Double stranded oligonucleotides containing the MCS were inserted between the BamHI and EcoRI sites. Three sets of oligonucleotides were used to create the three HA tags, each one being in a different frame as compared to the MCS, giving rise to pPRIG-Hind-HA 1, 2 and 3. The pPRIG-Hd was obtained by deleting the HA tag sequence after digestion with NgoAIV and BamHI, filling in with the Klenow polymerase and religation (the BamHI site is recovered in this process). All vectors from the pPRIG series are identical to the pPRIG-Hd vectors, except for the presence of the wild type EMCV IRES initiation codon in place of the HindIII site.

The pMigR vector [6] is similar to the pAP2-IRESeGFP yet differs in the following manner. The CMV promoter is replaced by an LTR. There are a few punctual differences in sequences in the LTRs as compared to the pAP2-IRESeGFP 3' LTR. The IRES sequence has conserved the 3' initiating AUG, which is mutated in the pAP2-IRESeGFP vector to generate a HindIII site. The eGFP polypeptide starts with its own AUG in the pAP2-IRESeGFP vector, while it is in frame with the IRES 3' AUG in the pMigR vector.

The pMigR-Hd, containing the pPRIG-Hd-HA-MCS and IRES (ATG mutated to generate a HindIII), was obtained by replacing the SpeI-NcoI fragment of the pMigR vector by the corresponding one from the pPRIG-Hd-HA. The pMigR-ATG, containing the pPRIG-Hd-HA MCS and the pMigR IRES (wt EMCV sequence retaining the 3' IRES ATG), was obtained by replacing the SpeI-DraIII of the pMigR by the corresponding SpeI/DraIII fragment from the pPRIG-HA vectors.

The EcoRI/Bsp120 I fragment of the pcDNA-BAZF vector encoding the mouse BAZF cDNA [10] was cloned at the EcoRI/NotI sites of the MigR-pAP7 HinDIII and MigR-pAP7 ATG vectors. The mouse AES (also known as GRG5) cDNA [11] containing the entire open reading frame was synthetized by PCR using retrotranscribed GS2 embryonic stem cell mRNA as templates. The PCR product (a gift from Dr D. Sekkaï) containing a 5' Xho I and a 3' EcoRI site brought by the primers, was cloned into the pDrive vector (Qiagen), verified by sequencing, and then cloned into the Xho I/Mfe I sites of the MigR-pAP7 HinDIII and MigR-pAP7-ATG vectors.

pPRIG-Hd-HA-Red, pPRIG-HA-Red and pAP2-HA-Red were obtained as follows. For, pPRIG-Hd-HA-Red and pPRIG-HA-Red, the BamHI-NotI fragment from pDsRED-N1 was cloned into pRIG-Hd-HA2 and pPRIG-HA2, respectively, digested with the same restriction enzymes. For pAP2-HA-Red, the NgoAIV-AccI fragment from pRIG-HA-Red was cloned into BBg (a derivative of pBluescript SKII containing a BglII site) digested with XmaI-AccI to give the BBg HA-Red from which the BglII -XhoI fragment (coding for the HA-DsRed fusion polypeptide) was purified and recloned into the pAP2-IRESeGFP vector opened by BglII and XhoI.

### Transfections/transduction

HEK 293T cells were grown in DMEM supplemented with 10% FCS. Cells were seeded at 30–40% confluence in 6 well plates (about 500 000 cells/well), and transfected the following day with 1.6 μg of the different expression vectors. Cells were analyzed between 24 and 72 h post-transfection. For virus production, the different expression vectors were cotransfected with 0.8 μg of pCMV-GagPol and 0.8 μg of pCMV-VSVG helper plasmids. Supernatants were collected 48 h post-transfection, passed through 0.45 μm filters, then added to the exponentially growing REF cultures in the presence of 4 μg/ml of polybrene. The medium was changed 16 h later, and transduced cells were kept in culture and passed 3× every 4 days.

### Western-blots

Cells were harvested, lysed and boiled in Laemmli buffer. 1/20 of each lysate was loaded and migrated by SDS-PAGE, and analysed by Western blotting. The membranes were incubated with F-7 mouse anti-HA antibody (1/500, Santa Cruz) and with rabbit A.v. peptide anti-GFP antibody (1/200, Clontech), followed by a peroxydase-coupled anti-mouse or anti-rabbit antibody, respectively. The signals were revealed by chemiluminescence.

### FACS analysis

48 h post- transfection, HEK 293T cells were rinsed and harvested in PBS, then analysed for GFP expression by flow cytometry using a Facscan (Becton Dickinson). 10000 events were recorded.

## Authors' contributions

MP carried out the molecular design and cloning of the pPRIG. OA carried out the FACS analysis and associated western experiment. MCP participated in the cell culture work and in western analysis. KEB participated in the culture work and in the drafting of the manuscript. PP participated in the design of the study, in its coordination, in the viral aspect of the work and in the writing of the manuscript. All authors have read and approved the final manuscript.

## Figures and Tables

**Table 1:** 


**Constructs**	**% GFP positive cells**	**Peak Value**
293T	0.05	1
pMigR	20.78	200
pMigR-Hd	15.8	14
pMigR-ATG	26.85	234
pMigR-Hd-BAZF	17.53	18
pMigR-ATG-BAZF	25.33	223

Top: Comparison of sequences flanking the initiation codon in high (Wild type IRES) and low (HindIII-modified IRES) IRES activity bicistronic vectors.

Bold font represent the original ATG EMCV IRES initiation codon, and the corresponding modified HindIII site. Italic font is part of the NcoI site (position 870 in the EMCV sequence) that can be used to clone the cDNA of interest under IRES control.

Bottom: FACS analysis of GFP expression of the different vectors.

HEK 293T cells were transfected with the indicated constructs, and subjected to FACS analysis 2 days post-transfection. The percentages of detected GFP positive cells are indicated, as well as the peak value of the GFP signals.

**Table 2:** 

Site	Compatible
**BamHI**	BglII/BclI
**PvuII**	Any Blunt
**EcoRI**	MfeI
**XhoI**	SalI/PspXI
**AvaIII**	PstI/Sse8387I
**ClaI**	AsuII/AccI (*)/AclI/AcyI/NarI
**AgeI**	Cfr9I (SmaI**)/BspMII/BetI/Cfr10I/NgoAIV (NaeI**)/SgrAI
**NotI**	Bsp120I (ApaI**)/CfrI/XmaIII
**SacII**	
**SplI**	Asp718I (KpnI**)/Bsp1407I/TatI
**StuI**	Any Blunt
**SphI**	NspI
**BspMII**	Cfr9I (SmaI**)/AgeI/BetI/Cfr10I/NgoAIV (NaeI**)/SgrAI
**Sse8387I**	PstI/AvaIII
**AsuII**	ClaI/AccI (*)/AclI/AcyI/NarI
**SalI**	XhoI/PspXI
**AccI**	ClaI/AsuII/AclI/AcyI/NarI
**HinDII**	Any Blunt
**BglII**	BamHI/BclI
**MfeI**	EcoRI

Polylinker structure.

Sites present in the polylinker, 5' to 3', are indicated in bold type. Compatible sites are shown on the right.

*: only when "MK" from AccI GTMKAC site is CG (K = G or T, M = A or C).

**: prototype site with distinct cleavage structure.
